# Testicular regression syndrome: A retrospective analysis of clinical and histopathological features in 570 cases

**DOI:** 10.3389/fped.2022.1006880

**Published:** 2022-10-31

**Authors:** Tian-Qu He, Rong Wen, Yao-Wang Zhao, Li Liu, Jian-Jun Hu, Yu Liu, Qian-Long Peng

**Affiliations:** ^1^ Department of Urology, Hunan Children’s Hospital, Changsha, China; ^2^ Department of Pathology, Hunan Children’s Hospital, Changsha, China

**Keywords:** cryptorchidism, testicular regression syndrome, testicular remnant, laparoscopy, germ cell

## Abstract

This study aimed to analyze the clinical features and pathological findings of the largest reported case series of testicular regression syndrome (TRS). Data, including age, affected side, color Doppler ultrasound results, surgical methods, intraoperative conditions, and pathological examinations, of children with unilateral TRS who were treated in our center from December 2012 to November 2021 were retrospectively analyzed. A total of 570 patients were included in this study. The mean age at surgery was 38 (range, 5–193) months. There were 457 cases (80.2%) of left TRS. Preoperative color Doppler ultrasonography found nubbins in 172 cases (30.2%). The long diameter of the contralateral testis was 17.11 (±4.22) mm, and the volume was 0.81 (±1.15) ml. The long diameter was ≥1.6 cm in 62.0% of the patients (240/387) aged ≤3 years. Laparoscopy was performed as the initial surgical step in 513 cases, of which 96.7% of the children had closed internal rings. One or more lesions of fibrosis, hemosiderin, and calcification were found in 92.4% (474/513) of the excised remnants. Germ cells were present in 16 cases (3.1%). In conclusion, TRS is more common on the left side and is usually accompanied by a closed internal ring and compensatory hypertrophy of the contralateral testis. Germ cells are only present in cases where the spermatic vessels enters the internal ring. We recommend that further exploration and excision of the remnants may not be applicable in cases where only the vas deferens has entered the internal ring.

## Introduction

Testicular regression syndrome (TRS), also known as vanishing testis, presents clinically as nonpalpable testis (NPT). A blind-ending spermatic vessels or the presence of a nubbin in which no normal testicular morphology can be identified (1–3) typically can be observed during surgical exploration. TRS accounts for 35%–60% of all NPT cases and usually needs to be differentiated from intra-abdominal and inguinal testes (2–5). Previous literature has suggested that compensatory hypertrophy of the contralateral testis with a spermatic vessels exiting a closed internal ring found by laparoscopic exploration is highly likely to indicate vanishing testis (4, 6–9).

The etiology of TRS remains unclear, although recent findings have been favored prenatal vascular accidents and testicular torsion (3, 10, 11). The histology of TRS specimens usually shows fibrosis, calcification, hemosiderin, testicular tissue, and paratesticular tissue (epididymis or vas deferens) (1–3). The incidence of germ cells (GCs) ranges from 0% to 16% in different studies, leading to controversy regarding the optimal treatment of TRS (1–3, 10, 12, 13). Some authors recommend its excision to eliminate the risk of potential malignancy (4, 10, 12). However, some researchers argue that excision is not necessary because of the low incidence of GCs and the rare probability of malignant transformation (1, 13). This study aimed to retrospectively analyze the clinical data and pathological results of 570 cases of unilateral TRS in our center in the past 9 years to provide a reference for the diagnosis and treatment of TRS.

## Materials and methods

We retrospectively studied unilateral TRS in 6,532 patients with cryptorchidism who underwent surgical treatment in our institution from December 2012 to November 2021. The inclusion criteria were blind ends of the spermatic vessels or a nubbin confirmed by surgical exploration. The exclusion criteria included cases of nonpalpable testis following an event of testicular torsion or inguinal hernia incarceration and those with a history of surgery in the inguinal-scrotal region.

Study-related data were collected through electronic medical record systems and pathology databases. Baseline data included age at the time of surgery, affected side, and preoperative color Doppler ultrasound results. The volume of the contralateral testis was calculated using the ellipsoid volume formula (π/6 × length × width × height). Intraoperative data included the surgical approach, the status of the spermatic vessels and vas deferens, and whether the internal ring was closed.

**Figure 1:**
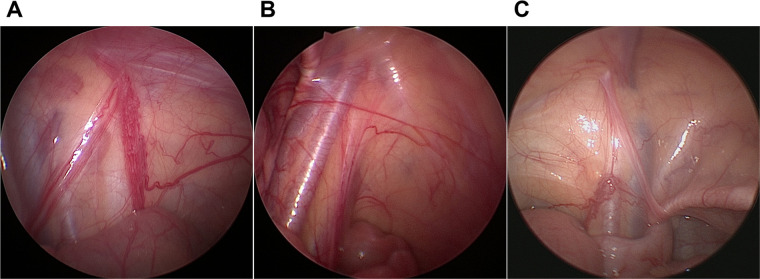
Laparoscopic findings in testicular degeneration syndrome. (**A**) Vas and vessels traversing the internal ring. (**B**) Blind-ending vas and vessels. (**C**) Blind-ending vessels with the vas deferens entering the internal ring.

Physical examination was performed again when patients underwent general anesthesia. If the testis could not be palpated, laparoscopy was used. If noticeable nubbins were palpable in the groin and scrotum, inguinal or scrotal exploration was adopted. Findings from diagnostic laparoscopy in patients with TRS were generally classified into the following three scenarios: the vas and vessels traversing the internal ring (Figure 1A); blind-ending vas and vessels at the proximal end of the internal ring (Figure 1B); and blind-ending vessels with the vas entering the internal ring (Figure 1C). The excision of the testicular remnant was performed *via* the surgeon's preferred approach (scrotal incision, inguinal incision, or excision of the distal remnant by retracting the spermatic vessels into the abdominal cavity). All excised remnant specimens were routinely stained with hematoxylin and eosin. Immunohistochemical staining was performed using anti-inhibin, Oct3/4, and Sall4 antibodies in cases where there were seminiferous tubules (SNTs).

Descriptive statistics were used to analyze the data. Enumeration data were expressed as *n* (%). Measurement data were expressed as mean (min–max) or mean (±SD).

## Results

**Figure 2:**
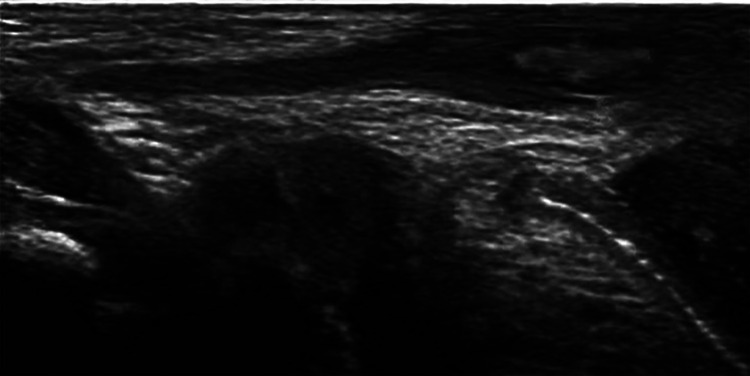
Ultrasound scan showing a hyperechoic nubbin located in the scrotum.

**Figure 3:**
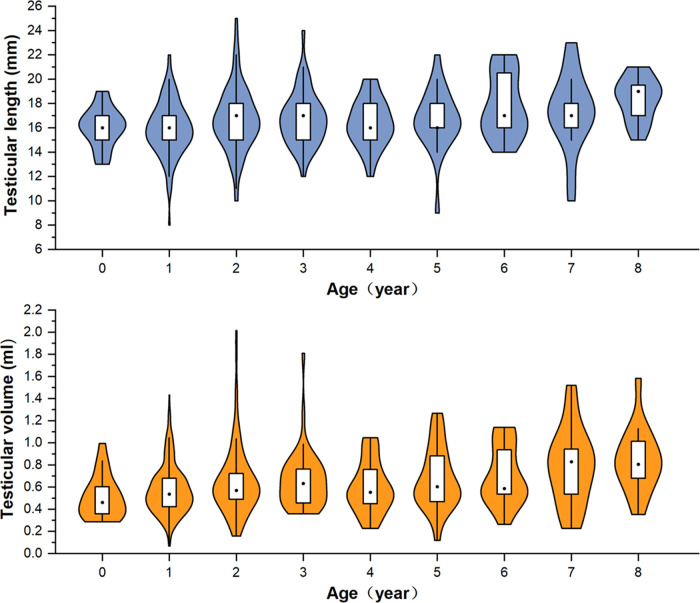
Violin-box plot showing the distribution and density of testicular length and volume in boys aged 0–8 years with monorchidism.

A total of 570 patients with unilateral TRS were enrolled in this study. The surgery was performed by 13 pediatric urologists from tertiary hospitals. The mean age of the patients at surgery was 38 (range, 5–193) months. There were 457 cases (80.2%) of TRS on the left side and 113 (19.8%) on the right side. Seventeen cases had contralateral cryptorchidism, of which four were located in the inguinal canal, three were in the superficial inguinal pouch, and 10 were of gliding testes. All cases underwent color Doppler ultrasonography before surgery. Color Doppler ultrasonography revealed nubbins in 172 cases (30.2%), of which 54 were located in the inguinal canal, 24 were in the inguinal region, and 94 were in the scrotum (Figure 2). Color Doppler ultrasound data showed that the length of the contralateral testis was 17.11 (±4.22) mm, and the volume was 0.81 (±1.15) ml. The long diameter was ≥1.6 cm in 62.0% of the patients (240/387) aged ≤3 years. The length and volume of the contralateral testis in patients aged 0–8 years are shown in Figure 3.

**Figure 4:**
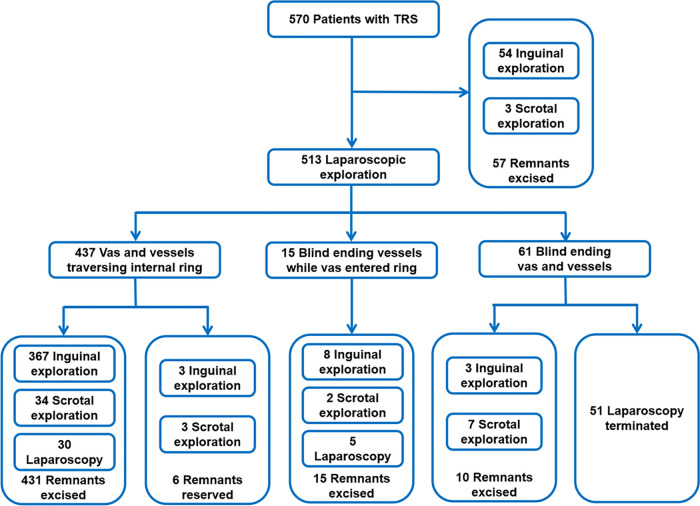
Overview of our approach and the surgical findings.

Laparoscopy was used as the initial surgical step in 513 cases. The internal ring was open on the affected side in 17 cases (3.3%), and the internal ring was open on the contralateral side in 51 (9.9%). The spermatic vessels and vas deferens traversed the internal ring in 437 cases (85.2%); their blind ends were found in 61 cases (11.9%), and only the vas deferens entered the internal ring in 15 (2.9%). Scrotal or inguinal exploration was performed as the initial step in 57 cases. The surgical approaches for all patients are shown in Figure 4. Patients with cryptorchidism or hydrocele on the contralateral side were treated concurrently during surgery, although the remaining patients did not undergo prophylactic orchiopexy of the contralateral testis. Follow-up was performed by telephone and outpatient review. Forty-five patients (7.9%) were lost to follow-up. The mean duration of follow-up for the remaining patients was 44 (range, 6–108) months, and no contralateral testicular torsion was reported.

**Figure 5:**
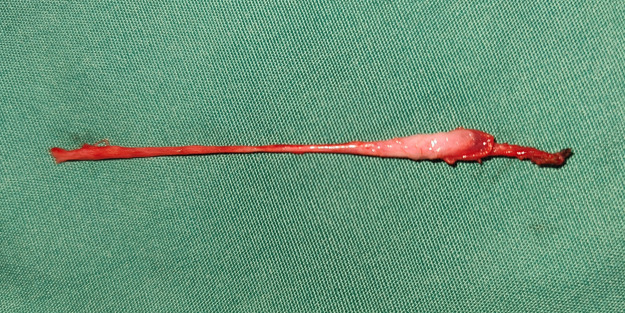
A testicular nubbin that was removed by laparoscopy, with the spermatic vessels and gubernaculum attached.

**Figure 6:**
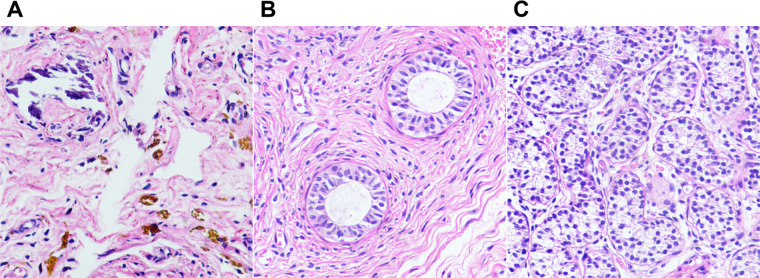
Photomicrographs of testicular degeneration syndrome. (**A**) Fibrous tissue with hemosiderin and calcifications (H&E, ×20). (**B,C**) H&E stains showing epididymal tissue (B, ×20) and seminiferous tubules (C, ×20) in the remnants.

**Figure 7:**
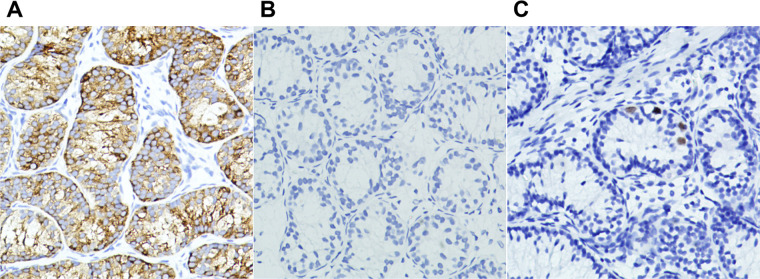
Photomicrographs of seminiferous tubules in testicular degeneration syndrome. (**A**) Sertoli-leydig cell population with immunohistochemical cytoplasmic positivity for inhibin (×20). (**B,C**) Immunohistochemical nuclear stains showing germ cells negative for OCT3/4 (B, ×20) and positive for Sall4 (C, ×20).

A total of 513 TRS remnants were excised (in six cases, the resection was not performed due to the guardian's refusal) (Figure 5). Hematoxylin and eosin staining demonstrated fibrosis in 460 cases (89.7%), calcification in 212 (41.3%), hemosiderin in 55 (10.7%), vas deferens in 351 (68.4%), epididymis in 228 (44.4%), and SNTs in 34 (6.6%) (Figure 6). One or more lesions of fibrosis, hemosiderin, and calcification were found in 92.4% (474/513) of the cases. Sertoli-stromal cells were positive for anti-inhibin in all cases with SNTs. GCs were nonreactive for Oct3/4 but were positive for Sall4 in 16 cases (Figure 7). Intratubular GC neoplasia and abnormal hyperplasia of GCs were not found in any cases. The findings from the intraoperative and pathological examination of the laparoscopic population are shown in Table 1.

**Table 1:** Summary of the intraoperative and pathologic findings in the laparoscopic population.

	Group 1	Group 2	Group 3
Vessels status	Entering	Blind ending	Blind ending
Vas status	Entering	Blind ending	Entering
Number of cases	437	61	15
Closed internal ring	420 (96.1)	61 (100)	15 (100)
Number of specimens	431	10	15
Fibrosis	388 (90.0)	10 (100)	14 (93.3)
Calcifications	184 (42.1)	4 (40)	3 (20)
Hemosiderin depositions	44 (10.2)	1 (10)	0 (0)
Vas deferens	295 (68.4)	1 (10)	9 (60)
Epididymis	187 (43.4)	0 (0)	2 (13.3)
Seminiferous tubules	26 (6.0)	0 (0)	0 (0)
Germ cells	12 (2.8)	0 (0)	0 (0)

Values are presented as *n* (%).

Entering: testicular vessels or vas deferens entering the internal ring. Blind ending: the testicular vessels or vas deferens appear to end before reaching the internal ring.

## Discussion

To the best of our knowledge, this study is the largest TRS cohort to date. Several theories have been proposed to explain the etiology of TRS, such as an underlying endocrine disorder, developmental interruption or failure of GC migration during the embryonic period, or regression and loss due to intra-abdominal location and temperature (2, 3, 5, 14). Nevertheless, recent research has favored the opinion of prenatal testicular torsion or other vascular accidents. Kraft et al. (11) performed a histological study of the contralateral testis with absent testis and cryptorchidism on the affected side, which uncovered differences in volume, GC counts, and pattern of maturation, suggesting that the etiology of TRS is more likely to be testicular torsion than endocrine disease. In our study, fibrosis, hemosiderin, and calcification are present in a large number of specimens, which implies the presence of hemorrhagic infarction in the testis during fetal life, also supporting the theory of vascular accidents (3, 10, 15). Left-sided TRS was more common in previous studies (66%–77.7%) (1, 4, 10, 12, 13, 15). In our study, the incidence of left-sided TRS was 80.2% (457/570). It is difficult to use endocrine factors to explain this significant laterality. Some authors have suggested that the anatomical relationship between the left testicular vein and left renal vein with an unusually mobile kidney that results in the twisting of the left testicular vein is a secondary cause of vascular occlusion (1, 12). In addition, earlier descent of the left testis during fetal life may also increase the risk of prenatal torsion.

The American Urological Association and European Association of Urology do not recommend the use of ultrasound as a preoperative test because of the associated low sensitivity and specificity for NPT (5, 16). However, in one survey, 49% of European urologists still implemented ultrasonography for NPT (17). The findings of Mori et al. (18) highlighted the assistance of ultrasound in the detection of testicular nubbins and indicated that the presence of hyperechoic areas in the mass and a testicular size ratio of less than 0.4 between the affected and unaffected sides might help identify testicular nubbins. Vos et al. (19) performed color Doppler ultrasonography in 96 patients with NPT. The results showed that ultrasonography has a high positive predictive value for the testis in the inguinal canal, which can help avoid unnecessary laparoscopic exploration. In our institution, all NPTs are routinely examined by sonographers, and the examination is relatively inexpensive (140 CNY, about 20USD). When the testis cannot be located on ultrasound, we emphasize to the parents of the patients the possibility of TRS or high cryptorchidism that requires staging surgery.

Mori et al. (18) found 34 testicular nubbins *via* color Doppler ultrasound, of which 23 were in the scrotum and 10 were in the inguinal canal. The ultrasonic results in our study revealed that 68.6% (118/172) of nubbins were outside the external inguinal ring. An anatomical study of 217 fetuses by Benzi et al. (20) showed that the inguinal-scrotal phase is a very rapid process, usually established in only 1–4 weeks. This helps explain why nubbins are more common near the scrotum. However, we did not include the locations of remnants in surgical records because procedures, such as the surgical approach and incision dissection, may have altered the initial location (13, 21).

Contralateral testicular hypertrophy (CTH) is a useful preoperative predictor of monorchidism (1, 11, 17, 22). A prospective study by Braga et al. (7) using calipers to measure the testis indicated that a contralateral testicular cutoff length of 1.9–2 cm had the best accuracy in predicting monorchidism in boys aged 11–30 months. However, ultrasound may achieve better precision than the orchidometer because it excludes interference from the epididymis, scrotal skin, and surrounding soft tissues (22). Furthermore, in our experience, boys with monorchidism in China have a shorter diameter of the testis, possibly due to the differences in geographic location, genetic susceptibility, environmental factors, and ethnicity (23). In the cohort of Gao et al. (1), 66.5% (143/212) of Chinese boys aged younger than 36 months with monorchidism had a contralateral testis of more than 1.6 cm in diameter. This proportion was 62.0% (240/387) in our cohort, in line with the findings of Gao et al. Although the findings of Mah et al. (17) indicated that CTH had no effect on NPT management decisions, we believe that this feature helps to reasonably reduce the parental expectation of the presence of a viable testis before surgery.

Findings in one survey suggested that 81%–97% of physicians would perform laparoscopy as the first step (17). They preferred to use laparoscopy to determine the absence of testes or treat an intra-abdominal testis (IAT), although an IAT is less likely to occur than an extra-abdominal (viable or remnant) testis (17). In this study, laparoscopy was used as the initial step in 90.0% of the cases. This procedure is also used in some cases of extra-abdominal nubbins found on preoperative ultrasound. The laparoscopic results of the absence of abdominal testes is a desired finding not only for parents, but also for surgeons, compared to the presence of nubbin-like tissue in the scrotum (8).

Some authors have indicated that the condition of the internal ring under diagnostic laparoscopy can aid in intraoperative decision-making (4, 6, 8, 9). The results from the study by Elder et al. (6) showed that 95.6% (43/45) of viable testes had patent processus vaginalis (PPV), compared with 5.1% (4/79) in the population with vanishing testis. Results of a recent prospective study showed that patients with absent testis were more likely to have a closed internal ring than patients with viable testis (4). In our previous study, 92.9% of the intra-abdominal and 72.9% of the inguinal testes had PPV (24). In the TRS cohort, this probability dropped to 3.3%. This is due to the fact that the proximal end of the processus vaginalis is usually closed after the testis descends, and prenatal testicular torsion usually occurs after this process (6).

In previous studies, the phenomenon that only the vas deferens enters the internal ring is less mentioned. The testis and epididymal vas deferens evolve from the sex cord of the gonadal ridge and mesonephric duct, respectively, during the process of embryonic development (25). In contrast, the epididymis, vas deferens, and seminal vesicles originate from the Wolffian duct under the influence of testosterone produced by Leydig cells (14). The study by Bouzada et al. (26), based on human anatomy and embryology, showed that the proximal end of the gubernaculum is attached to the epididymis and vas deferens and that the epididymis is usually positioned lower than the testis during testicular descent. In severe cases of testicular epididymal dissociation, the testis does not descend with the epididymis but remains in the abdominal cavity (25). These results help explain our laparoscopic findings. Furthermore, SNTs were absent in histological specimens from this subgroup previously reported in the literature, which was consistent with our findings (8, 9, 21). Therefore, we believe that this kind of case does not need further exploration.

In our cohort, none of the patients underwent prophylactic orchiopexy of the contralateral testis during the operation, and none developed testicular torsion in the follow-up population. Kehoe et al. (2) suggested that prophylactic orchiopexy of the contralateral testis should be abandoned when testicular nubbins are found because the risk of torsion is the same as that in the general population. The results of the study by Martin et al. (27) revealed that the incidence of Bell clapper anomaly in the contralateral testis of monorchidism and older boys with acute testicular torsion was 2% and 78%, respectively, and the extremely low torsion probability of the former ones is insufficient to support conventional orchiopexy. The long-term fertility potential of boys with monorchidism is also a concern. The findings from the study by Kraft et al. (11) suggested that, unlike cryptorchidism, unilateral TRS may not affect fertility. This finding is supported by another study that found no reduction in paternity among patients with monorchidism compared with the general population (28). However, corresponding counseling should be provided for patients because any damage to the remaining testicle would be catastrophic.

The main issue in the current debate on TRS is whether or not the testicular remnant needs to be excised. Different GC incidences reported in the literature may be the reason for this divergence (2, 3, 10, 12, 13). The results of the study by Woodford et al. (15) showed that there was no viable GC in the testicular nubbins. Nonetheless, the histopathological study of 332 TRS cases by Gao et al. (1) indicated that 21 (6.3%) had SNTs and seven (2.3%) had GCs. A recent meta-analysis showed that the incidence of GCs ranged from 0% to 15.4%, with an overall incidence of 5.3% (13). The differences in the incidences of GCs may be attributed to the different histopathological methods and antibodies adopted by the researchers. We used two antibodies to target GCs, although all specimens were negative for Oct3/4. The study by Antic et al. (14) confirmed the normal regulation of Oct3/4 antigen expression in the testis from reactivity in fetal life to nonreactivity in postfetal life. A total of 16 (3.1%) GCs were labeled by Sall4 in this study, which was consistent with that in previous studies (1, 13). To date, only one case of intratubular GC neoplasia in testicular remnants lacking immunohistochemical support has been reported (29). The low incidence of GCs and extremely rare probability of malignancy predispose the controversy to the opinion that excision of the remnant is unnecessary, which overlooks surgical procedures in clinical practice from our perspectives. The main purpose of surgical exploration for NPT is to determine the presence or absence of a viable testis, which can only be achieved by the exposure of the ends of the spermatic vessels, in the context of which the excision does not add much operating time. As mentioned above, we believe that in cases where only the vas deferens entered the internal ring and further exploration may not be necessary, mainly because the presence of an extra-abdominal testis is unlikely in this kind of case. In addition, Pathak et al. (30) reported two cases of testicular nubbins where ectopic adrenal cortical tissue was found, and they advocated routine excision of testicular remnants considering the risk of long-term malignant transformation of adrenal cortical tissue.

Our study has some limitations. First, there was inconsistency in surgical decision-making and techniques among various surgeons. Second, a control group of NPT cases with viable testes for further analysis of the predictive effect of clinical features on TRS was lacking. Finally, there was also a lack of a reliable control group to study the benefits and risks of not excising the remnant. Further research to address these limitations would be valuable.

In conclusion, TRS is more common on the left side and is usually accompanied by a closed internal ring and compensatory hyperplasia of the contralateral testis. GCs are only present in cases where the spermatic vessels enters the internal ring. Therefore, we recommend that further exploration and excision of the remnants may not be applicable in cases where only the vas deferens has entered the internal ring.

## Data Availability

The raw data supporting the conclusions of this article will be made available by the authors, without undue reservation.
